# Asian trends in primary androgen depletion therapy on prostate cancer

**DOI:** 10.7497/j.issn.2095-3941.2013.04.002

**Published:** 2013-12

**Authors:** Hideyuki Akaza

**Affiliations:** Department of Strategic Investigation on Comprehensive Cancer Network, Research Center for Advanced Science and Technology, The University of Tokyo, Tokyo 113-8654, Japan

**Keywords:** Androgen depletion therapy, prostate cancer, Asia

## Abstract

There are notable differences in the incidence and mortality rates for prostate cancer between Asia and Western countries. It is also recognized that there are differences in thinking with regard to treatment options. Recently it is also the case that opinions have been reported concerning the differences between Asian and Western patients with regard to their reaction to androgen depletion therapy (ADT). Given that ADT is a method of treatment that focuses on the elimination of testosterone, an inevitable symptom of its administration is testosterone losing syndrome. It is for this reason that in Western countries ADT has only been recommended in cases of advanced or metastatic cancer. On the other hand, in Asia, ADT is used in relatively many cases, including non-metastatic localized cancer and invasive localized cancer. To date, however, there has been little substantive discussion concerning this difference in utilization of ADT. ADT-related drugs for prostate cancer and the development of new drugs for castration resistant prostate cancer (CRPC) have been actively tested in recent years. It could be the case that analyzing the differences in concepts about ADT between Asia and the West could contribute to the effective use of ADT-related drugs and also help to build new treatment strategies for prostate cancer.

## Introduction

It is well known that there are significant regional differences in the incidence and mortality rates of prostate cancer. The difference is particularly notable between the Asian region and European countries. Prostate cancer is an androgen sensitive cancer and responds well to androgen depletion therapy (ADT), however, one of the unique drawbacks to this treatment is that it causes testosterone losing syndrome. It is for this reason that in Western countries the role of ADT in treating prostate cancer is generally limited to metastatic cancer and incurable advanced-stage cancer. On the other hand, in many of the countries in Asia, including Japan, ADT has for many years been relatively often used also for localized cancer, which perhaps reflects the social and philosophical background of Asia.

However, comparative studies of the outcomes of ADT in Asia and the West have actually only recently been initiated, and the clinical significance of ADT in both Asia and the West remains unclear.

In this paper the history of a unique joint collaborative study in Asia on ADT will be introduced and an overview of a registry study, which has developed out of previous efforts, will also be introduced. The significance of ADT for prostate cancer treatment will be discussed.

## The history of Asian collaborative study of ADT on prostate cancer (Table 1)

**Table 1:** Conference of Asian trends in prostate cancer hormone therapy

Conference	Place	Data
1^st^	Singapore	September, 2001
2^nd^	Hong Kong	December, 2002
3^rd^	Tokyo	December, 2003
4^th^	Honolulu	October, 2004
5^th^	Bali	August, 2006
Korea-Japan	Seoul	September, 2005

An international conference titled “Asian trends in prostate cancer hormone therapy” was first held in 2001, with committee members comprising primarily urology specialists from a number of regions, including Japan, China, Korea, Singapore, Indonesia and Taiwan. The first conference in 2001 was held in Singapore, followed by the second in Hong Kong (2002), third in Tokyo (2003), fourth in Honolulu (2004), and the fifth in Bali, Indonesia (2006)^1^^-^^4^. At the fifth conference 27 uro-oncologists from Asia participated. This conference was also attended by Dr. Malcolm Moore, inaugural director of the Union for International Cancer Control (UICC) Asia Regional Office (ARO), who emphasized the necessity for the construction of a registration system and the importance of screening, prevention and diet control^5^.

Similar meetings were also held in 2005 on a bilateral basis between Japan and Korea. These various approaches were discussed and brought together at a Prostate Cancer Working Group meeting held at the 20^th^ Asia-Pacific Cancer Conference (APCC) in Tsukuba, Japan in 2010^6^.

Through the course of these various meetings, a wide-ranging discussion took place on the current status of ADT for prostate cancer treatment in Asia and the various issues being faced overall. At the first meeting data was collected concerning the patient characteristics of prostate cancer patients in Asia, including the proportion that was administered ADT at various stages of cancer. Although the scale of data sources differed from country to country, the proportion of patients being administered ADT as an initial therapy at each cancer stage was compared, and it was shown that at each cancer stage ADT was selected as a therapy for a relatively large proportion of patients^1^. At the second meeting, in an attempt to uniformalize data sources, 100 recently diagnosed new patients were registered from among participating member institutions and a comparison was implemented. In the comparison of the cancer stage of each patient at which ADT was administered as an initial therapy, with the exception of Singapore, all other countries reported that there were many cases in which ADT was selected for localized prostate cancer. In the case of Singapore, it was mainly T_4_ patients who received ADT^2^. At the third meeting a comparison of quality of life was implemented, using the same method as before, with 100 registered patients from the various countries. The same questionnaire was issued to patients for comparison purposes. Furthermore, at the third meeting discussion took place concerning the number of prostate biopsy cores and also on the need for and feasibility of implementing a joint multi-institutional study in Asia^3^. In the Prostate Cancer Working Group that was organized as part of the APCC, discussion took place concerning the low incidence of prostate cancer in Asia and the relationship between this low incidence and the large consumption of soy-based food products. In addition, discussion also focused on a comparison between Asia and the West with regard to the significance of ADT and on the necessity to establish and interpret unique guidelines for ADT in Asia. The conference on Asian trends in prostate cancer hormone therapy was ultimately disbanded due to a lack of operating funds. However, the spirit of that conference remains with us today, in the form of the Asia-Pacific Prostate Society^7^.

## Asian consensus statement for NCCN clinical practice guidelines of prostate cancer

The National Comprehensive Cancer Network (NCCN) clinical practice guidelines are used widely and frequently in all regions of the world, not just in the cancer center in the United States where they originated. They have become the standard for cancer treatment. However, given the various conditions in each country and region, it does not necessarily mean that all medical institutions can provide treatment that is exactly in accordance with the guidelines. In particular, various factors in Asia, including those of a social, economic, philosophical, medical and religious nature, are obstructing implementation in accordance with the guidelines. The compilation of any guideline should be implemented on the basis of medical evidence, using cohort studies, and the situation in Asia is that there is still a lack of such medical evidence, meaning that the only option is to utilize the global guidelines as set forth by the NCCN.

The Asian consensus statement (ACS) for NCCN clinical practice guidelines takes these various constraints into consideration and represents the results of discussion about how the countries of Asia can implement treatment that matches the conditions in each particular country. In the field of urological malignant tumors, the ACS is being followed in the two areas of renal cancer and prostate cancer^8^^,^^9^.

From these efforts it has become apparent that the method of use of endocrine therapy for prostate cancer is different in Asia compared to the West. In other words, in Asia, even in cases where prostate cancer has been diagnosed at a relatively early stage, the use of primary androgen depletion therapy (PADT) is being promoted. In September 2013 ACS committee meeting for NCCN clinical practice guideline for prostate cancer was held in Inchon, Korea under the auspice of Asia Pacific Prostate Society, Asia Pacific Society of Uro-oncology and Japanese Society of Clinical Oncology.

## Importance of registry studies

Clinical trials tend to have stringent entry criteria. Even patients who are eligible to enter a clinical trial may not be willing to participate because of the more extensive study procedures, the risk of not receiving the medication they want or for a host of other reasons. These factors significantly limit the generalizability of the study results to the general population.

Registries mitigate these limitations by casting a wider net to include a wider range of patients. Thus, results of registry studies are closer to real-world situations and have greater generalizability. Two such important long-term, large-scale, longitudinal observational databases on prostate cancer are J-CaP (Japan Study Group for Prostate Cancer) from Japan and CaPSURE (Cancer of the Prostate Strategic Urologic Research Endeavor) from the United States.

J-CaP surveillance is a nationwide longitudinal observational study of patients newly starting hormone therapy for prostate cancer from January 2001 to December 2003 with more than 26,000 cases enrolled. CaPSURE on the other hand, was initiated in 1995 to document national trends in prostate cancer epidemiology, disease management, oncologic outcomes, and health-related quality of life (HRQOL) outcomes. It is a longitudinal and observational database accruing data from a total of 40 urologic practice sites over the history of the registry. It currently has around 14,000 patients in their database.

A joint initiative was established in 2007 with the objective of analyzing, reviewing, comparing and contrasting data from J-CaP and CaPSURE registries. This comparison has shown many similarities as well as differences in the treatment and treatment outcomes of prostate cancer between Japan and US^10^^,^^11^.

## Effect of PADT and different outcomes between Japan and the United States

PADT is endorsed as an option for monotherapy for localized prostate cancer by guidelines in Asia but not in the U.S. or Europe^12^. PADT monotherapy is commonly used, however, in both the U.S. and Japan, especially for high risk groups in the U.S. In other Asian countries it is also commonly used^11^.

Data were analyzed from the CaPSURE registry representing community-based practice in the U.S., and from the J-CaP database. Risk adjustment was performed using the Japan Cancer of the Prostate Risk Assessment (J-CAPRA) score, validated specifically for men with advanced disease and those treated with PADT^13^ (**Table 2**). Prostate cancer-specific mortality (PCSM), adjusting for age, J-CAPRA, year of diagnosis, and treatment type [combined androgen blockade (CAB) *vs.* medical or surgical castration monotherapy] were analyzed. Men treated with PADT in J-CaP were slightly older than those in CaPSURE, and had a higher risk disease. They were more likely to be treated with CAB. In the multivariable regression analysis, the hazard ratio (HR) for PCSM was 0.31 for J-CaP compared to CaPSURE. In J-CaP, CAB improved survival compared to castration monotherapy, but this effect was not observed in CaPSURE. For all-cause mortality, the HR for J-CaP was 0.27.

**Table 2:** Risk assessment: J-CAPRA^13^

Variable	Level	Points
PSA	0-20	0
	20-100	1
	100-500	2
	>500	3
Gleason	2-6	0
	7	1
	8-10	2
T-stage	T_1a-2a_	0
	T_2b-3a_	1
	T_3b_	2
	T_4_	3
N-stage	N_1_	1
M-stage	M_1_	3

Sum of points from each variable for 0-12 score.

Adjusting for multiple factors including disease risk and type of androgen ablation, men treated with PADT in Japan compared to the U.S. have greater than three-fold better cancer-specific survival and four-fold better overall survival. CAB improves outcomes compared to castration monotherapy in J-CaP but not in CaPSURE^14^. The report concluded that these findings substantiate guidelines both encouraging PADT in Asia and discouraging its use in the U.S. The reasons for these substantial differences are likely multifactorial, including both genetic and environmental factors, and elucidating them will likely yield critical insights into the biology of prostate cancer on both sides of the Pacific.

## Intermittent androgen depletion (IAD) therapy and continuous androgen depletion (CAD) therapy

CAD therapy is the standard treatment for metastatic prostate cancer. CAB is the regimen most often used in Japan^15^. On the other hand, IAD therapy allows for testosterone levels in the blood to be recovered during the periods when it is not being administered, which contributes to improving quality of life (QOL). In addition, animal tests have suggested the possibility that IAD could delay the advance of CRPC^16^. In recent years ADT has come to be often used in cases of non-metastatic prostate cancer^10^, and in such cases there are expectations that IAD could prove to be of particular benefit. The results of a randomized clinical trial (RCT) implemented by Crook *et al.*^17^ could be said to be representative of the expectations for IAD. At the same time, at the annual meeting of the American Society of Clinical Oncology (ASCO) in June 2012, the results of a long-term, large-scale RCT into IAD for metastatic prostate cancer were announced. These results provided important new information about the effect of IAD and created considerable discussion^18^^,^^19^.

## Benefits of IAD

According to the NCCN Clinical Practice Guidelines for prostate cancer (2013 v.4), “Intermittent ADT may reduce side effects without altering survival compared to continuous ADT, but the long-term efficacy of intermittent ADT remains unproven.”^12^. To date a number of RCT have compared IAD and CAD and have shown that there are no differences in overall survival time^20^, however, the observation period (median value) ranges from 30.8 months to approximately 6.9 years and therefore cannot be said to be sufficient. The SWOG 9345 (INT-0162) trial reported by Hussain *et al.*^19^ was a large-scale, long-term test with a median observation period of 9.2 years, involving eligible cases from a total of 1,535 patients. To date this has been the study from among the published RCT that have compared IAD and CAD that has had the longest observation period and largest number of cases. As noted above, the RCT by Crook *et al.*^17^ was a comparative study on non-metastatic prostate cancer and contrasted with the SWOG 9346 trial.

In this paper the results of two recent representative RCT that sought to compare IAD and CAD have been cited. It is believed that these two papers will continue to be cited in the future as important RCT in terms of both their scale and quality. At the same time, these trials examined cases of PSA relapse after radiotherapy in localized prostate cancer and also the uses of ADT for metastatic prostate cancer.

So is the use of IAD a good or a bad thing?

To sum up, IAD is linked to individualization of treatment. This may be simplistic, but at the current point this is all that can be said. The history of treatment of prostate cancer is relatively long and it has various aspects, depending on staging at the time of diagnosis and the pathological background. Although various RCTs have been implemented, it is virtually impossible to arrive at a universal conclusion with regard to the use of IAD.

This is because it is necessary to consider and deal with countless confounding factors, including patient background, therapeutic drugs, dosage, timing of the start of administration, timing of halting of administration, establishment of endpoints for trials, and balancing QOL with treatment costs, etc.

So why are such complex RCTs necessary?

The answer to this question is deeply linked to the fact that even today more than 70 years since Dr. Huggins first proposed ADT^21^, the basic drug therapy for prostate cancer is elimination of testes-derived testosterone. In other words, to the extent that this drug therapy is all that is available to us, we will be unable to break away from the curse of testosterone losing syndrome. The major significance of IAD is that it helps to support QOL and therefore more large-scale RCTs on IAD will be required in the future.

Based on recent results of androgen-axis research, we cannot yet allow ourselves to believe at any time soon that drug therapy for prostate cancer will be free from the fixed notion that the principle for treatment is testosterone elimination.

If it were possible to eliminate the clinical challenges presented by testosterone losing syndrome, the significance of comparing IAD and CAD would also virtually disappear.

## Future concept of ADT

Prostate cancer is a classic androgen-sensitive cancer. The effectiveness of ADT at all clinical stages of prostate cancer is clear and significant. In other words, if testosterone is eliminated it is possible to easily control the disease. However, there are two significant issues that face ADT for prostate cancer. Firstly, ADT is not a radical treatment. Secondly, current ADT focuses on the elimination of testosterone. With regard to the first issue, a body of knowledge has been built up for ADT with regard to advanced cancer and metastatic cancer. Within these categories it is known that over the course of a number of years ADT becomes ineffective against many forms of prostate cancer. However, the fact that new drugs for CRPC have shown clinical effectiveness suggests that current ADT does not completely eliminate testosterone or completely inhibit the action of testosterone androgen receptors. These facts imply that in the future it will not be impossible to establish more powerful 1^st^ line ADT. Furthermore, the possibilities for pathway control using AR receptors suggest that the adverse effects of testosterone elimination, namely testosterone losing syndrome, could be avoided in the future. It is believed that the role of ADT in prostate cancer treatment will become increasingly important.
